# Effects of work-related factors on the breastfeeding behavior of working mothers in a Taiwanese semiconductor manufacturer: a cross-sectional survey

**DOI:** 10.1186/1471-2458-6-160

**Published:** 2006-06-21

**Authors:** Yi Chun Chen, Ya-Chi Wu, Wei-Chu Chie

**Affiliations:** 1 School of Nutrition and Health Sciences, Taipei Medical University, 250 Wu-Hsing Street, Taipei 110, Taiwan; 2 Division of Clinical Sciences, Center for Drug Evaluation, 1F, No15-1, Sec. 1, Hangjou S. Rd., Taipei 100, Taiwan; 3 Department of Public Health and Institute of Preventive Medicine, College of Public Health, National Taiwan University, room 520, No.17 Xu-Zhou Road, Taipei, 100, Taiwan

## Abstract

**Background:**

In recent years, the creation of supportive environments for encouraging mothers to breastfeed their children has emerged as a key health issue for women and children. The provision of lactation rooms and breast pumping breaks have helped mothers to continue breastfeeding after returning to work, but their effectiveness is uncertain. The aim of this study was to assess the effects of worksite breastfeeding-friendly policies and work-related factors on the behaviour of working mothers.

**Methods:**

This study was conducted at a large Taiwanese semiconductor manufacturer in August-September 2003. Questionnaires were used to collect data on female employees' breastfeeding behaviour, child rearing and work status when raising their most recently born child. A total of 998 valid questionnaires were collected, giving a response rate of 75.3%.

**Results:**

The results showed that 66.9% of survey respondents breastfed initially during their maternity leave, which averaged 56 days. Despite the provision of lactation rooms and breast pumping breaks, only 10.6% mothers continued to breastfeed after returning to work, primarily office workers and those who were aware of their company's breastfeeding-friendly policies.

**Conclusion:**

In conclusion, breastfeeding-friendly policies can significantly affect breastfeeding behaviour. However, an unfavourable working environment, especially for fab workers, can make it difficult to implement breastfeeding measures. With health professionals emphasizing that the importance of breastfeeding for infant health, and as only females can perform lactation, it is vital that women's work "productive role" and family "reproductive role" be respected and accommodated by society.

## Background

In recent years, the creation of supportive environments for encouraging mothers to breastfeed their children has emerged as a key health issue for women and children. While the number of new mothers in the workplace increases, an early return to work and inconvenient workplace conditions discourage women from breastfeeding or cause them to discontinue breastfeeding early [1-3].

The World Health Organization (WHO) recommends exclusive breastfeeding for the first six months of life [4]. The length of maternity leave is positively associated with the duration of breastfeeding [5,6]. The International Labour Organization (ILO) recommends a period of maternity leave of not less than 14 weeks [7]. However, the typical maternity leave in many Asian and Middle Eastern countries falls below these levels, only offering less than 12 weeks paid leave [8]. In Taiwan, most companies provide only eight weeks of maternity leave. A national survey in 2005 showed that the rate of exclusive breastfeeding in Taiwan at one month postpartum was only 22.3%, and dropped to 16.7% at three months [9]. To bring Taiwan in line with WHO guidelines, effective worksite strategies needed to be implemented to encourage new mothers to breastfeed in the workplace.

The provision of lactation rooms and breast pumping breaks have helped mothers to continue breastfeeding after returning to work, but their effectiveness is uncertain. This study examines breastfeeding policies in a large Taiwanese semiconductor manufacturer. The semiconductor industry is key growth river of Taiwan's economy, and also plays a significant role in the economies of many other developing and newly industrialized countries. Thus, the breastfeeding needs of female workers in Taiwan's semiconductor companies are very important and similar to such countries. The study also examines work-related factors that affect women's breastfeeding behavior.

The purpose of the study is to conduct a survey of new mothers in the workplace to explore the correlation between breastfeeding and work barriers to achieving WHO recommendations.

## Methods

### Research setting and subjects

The research setting is Company T, a large semiconductor manufacturer with 10,000 employees in Hsinchu Science Park in northern Taiwan, which is one of the world's most significant areas for semiconductor manufacturing, similar in importance to Silicon Valley. This company was selected because it was the first semiconductor manufacturer to provide lactation rooms and breast pumping breaks for working mothers, which was an uncommon practice at the time the survey was conducted. Secondly, about 50% of the company's employees are women, most of whom are of childbearing age. Thirdly, the female employees are office workers or fab workers or, so the influence of different working conditions on breastfeeding behavior could be observed.

Office workers had superior educational levels and higher compensation levels compared with the fab (fabricating) workers, and they worked about eight hours a day from 08:30 to 18:00. Their positions encompassed specific job responsibilities, and so their bonuses were largely tied to individual performances. By comparison, fab workers worked 12-hour shifts, from 07:00 to 19:00, or from 19:00 to 07:00. Their jobs were inconvenient and inflexible, as they have to remove and put back on their clean room suits when leaving and returning to their workstation, and a substitute had to take their place during their absence. Performance bonuses at most Taiwanese manufacturers are based on both group productivity and individual performance criteria. It is the group leader's responsibility to promote higher productivity as well as evaluate individual performances. If productivity is very high, a worker's annual bonus can usually exceed their base salary. Therefore, most workers will do their best to maximize productivity.

### Data collection and variable definitions

This study was conducted between April and September 2003. Questionnaires were used to collect data on female employees' breastfeeding behaviour, child rearing and work status when raising their most recently born child (See Additional file 1). In developing the questionnaire, in-depth interviews were conducted with 25 female workers at Company T. Seven public health professionals also reviewed and revised the questionnaire. The preliminary questionnaire was further revised after it was used in a pilot study at another semiconductor manufacturer. The final questionnaire was then distributed in August and September 2003 to 1,326 female employees, whom had taken maternity leave between January 1999 and April 2003 as recorded by the manufacturer's human resource department. The company's occupational and environmental health nurses helped distribute and collect the questionnaires. To help clarify questionable answers, the questionnaire was not anonymous, but all personal information and answers were kept confidential by the researchers. Written consent was not required in questionnaire survey. Participation in this study is entirely voluntary. We described the aims of this study and invited the subjects to participate in this study, and oral consent was obtained from the participants. This study was reviewed and approved by the ethics committee of the College of Public Health, National Taiwan University.

The study used two dependent variables: breastfeeding initiation (whether or not the mother had ever breastfed the most recently born child) and continuation of breastfeeding after returning to work (yes or no). A mother initiated breastfeeding if she had ever breastfed her child. Mothers were defined as breastfeeding only during maternity leave (initiation and no continuation) if they completely stopped breastfeeding before, or within two weeks after returning to work. Mothers were defined as continuing breastfeeding if they continued for at least two weeks after returning to work.

Several employment characteristics were analyzed as independent variables, including worksite (fab/office), according to human resource department records), shift work ("Did you do shift work after you returned to work?" yes/no), flextime ("Did you need any substitute when you left your position during working hours? " yes/no), child's age (< 1 year, 1–3 years or >3 years) and length of employment (≤ 6 years, 7–9 years or ≥ 10 years). Other independent variables were an employee's knowledge of breastfeeding policies in the workplace, including awareness of lactation rooms ("Did you know about the lactation room in your company?" yes/no) and awareness of breast pumping breaks. ("Did you know about pumping breaks? " yes/no).

### Statistical analysis

The effects of employment characteristics and manufacturer policies on breastfeeding behavior were estimated using chi-square tests and logistic regression. Preliminary associations between categorical variables were verified through chi-square tests on contingency tables. Logistic regression was used to analyze breastfeeding behavior with respect to the following variables: age, education, worksite, shift work, years of employment and policy awareness. Variables were culled after an evaluation of their collinearity. If two or more variables were highly correlated, separate models were run for each of the collinear variables, and the model with the strongest relation to the breastfeeding measures was selected. Statistical analyses were conducted using SAS Version 8.2.

## Results

### Demographic and social characteristics of respondents

A total of 998 valid questionnaires were collected, giving a response rate of 75.3%. As shown in Table 1, the mean age of the mothers was 29.8 years, and the mean maternity leave was 56.8 days. About half of the women had either a high school degree (51.6%) or a college degree (48.4%). 43.3% were first-time mothers and 50.9% of newly borns were male. Most worked in the semiconductor fab (82.1%), were shift workers (77.2%), and their work time was inflexible (70.6%). The breastfeeding initiation rate was 66.9%, but 62.5% of breastfeeding mothers only breastfed less than one month.

### Breastfeeding status

Breastfeeding behavior was grouped according to socio-demographic and working characteristics, as shown in Table 2. 56.3% only breastfed during maternity leave, and 33.1% never breastfed. Despite the provision of lactation rooms and breast pumping breaks, only 10.6% continued to breastfeed after returning to work. All independent variables were significantly correlated to breastfeeding behavior (p < 0.001). Older age, lower education, fab work, shift work, inflexible schedule, and not knowing about worksite breastfeeding policies were all associated with the discouragement breastfeeding initiation. Of those who did not know about the lactation rooms, 47.2% never breastfed their last child. A child's age and years of employment were negatively correlated to breastfeeding initiation. Almost half of women whose children were more than three year's old, and 49.7% of those who had worked for the company for ten or more years, did not initiate breastfeeding.

Older age, higher education, less than one-year old child, office work, non-shift work, flexible schedule and knowing about worksite breastfeeding policies were all associated with the increased likelihood of a mother continuing to breastfeed after returning to work. 29.2% of office workers continued to breastfeed. Of those who knew about lactation rooms, 13.5% continued to breastfeed, whereas 20.1% of those who knew about breast pumping breaks continued to breastfeed. Mothers who were employed for ten or more years had the lowest continuation rate.

### Multivariate logistic regression analysis

Table 3 shows the results of the logistic regression analysis. Shift work was excluded from the models because it was highly correlated with the worksite location (γ = 0.83). Flextime was excluded because it had little or no effect on the models. Age, education, worksite, years of employment and awareness of lactation room were significantly correlated with breastfeeding initiation. Only the child's age, worksite location, awareness of lactation rooms, and awareness about breast pumping breaks were significantly correlated with continued breastfeeding. The odds ratio (OR) of continuing breastfeeding was 2.53 (95% CI, 1.55–4.14) for child aged <1 year old and 1.64 (95%CI, 1.10–2.42) for child aged between 1 and 3 years. The OR of continuing breastfeeding was 4.32 (95%CI, 2.30–8.11) for office workers, 2.71 (95%CI, 1.19–6.15) for women who knew about the lactation rooms and 2.68 (95%CI, 1.57–4.58) for women who knew about breast pumping breaks.

### Awareness of policy and worksite

Table 4 shows the different effects of breastfeeding policy awareness between office and fab workers. Workers who knew about the lactation rooms were significantly more likely to initiate breastfeeding. The OR of initiating breastfeeding was 7.88 (95% CI, 2.36–26.32) for fab workers and 2.83 (95% CI, 0.99–8.06) for office workers. Policy awareness had a stronger effect on continuing breastfeeding for office workers than for fab workers. For fab workers, only awareness of breast pumping breaks was significantly related to continuing breastfeeding (OR = 2.15, 95% CI, 1.09–4.26). For office workers, the OR for continuing breastfeeding was significantly higher for those who knew about the lactation rooms (OR = 6.53, 95% CI, 1.33–32.13) and those who knew about the breast pumping breaks (OR = 4.91, 95% CI, 1.79–13.46). For those women who knew about both the lactation rooms and breast pumping breaks, there was a higher probability that office workers would continue breastfeeding (OR = 8.65, 95% CI, 2.48–30.18).

## Discussion

### Breastfeeding status

In this study, the average breastfeeding rate was 66.9%. It was much higher than the national average of 22.3% at one month postpartum [9]. However, the rate of breastfeeding after returning to work was only 10.6%, much lower than the national average of 16.7% at three months postpartum [9]. Furthermore, the rates differed among groups. Younger mothers were more likely than older women to breastfeed, but most of them only breastfed during maternity leave. Most of the younger mothers worked in the fab plant and so may found it difficult to use the breast pumping breaks and lactation rooms, implying that an inconvenient working environment is an important barrier to breastfeeding among working mothers. Mothers with children less than three years old were more likely to continue breastfeeding than those with older children. This may not only because the memory recall effect but also the effect of pro-breastfeeding governmental policy and social environment in recent years.

### Importance of policy

The study found that awareness of breastfeeding-friendly measures in the workplace can significantly increase continued breastfeeding rate after returning to work. But as breastfeeding after returning to work is still a new practice in Taiwan, and the target company has made considerable efforts to establish and promote this policy, the possibility of reverse causality is not very high. It is important to encourage industries to provide breastfeeding support services, such as providing breast pumping breaks or lactation rooms. Previous research has shown that work is one of the biggest obstacles to breastfeeding [10-12]. Though breastfeeding and employment are not mutually exclusive[13], longer periods of maternity leave may be a good solution to promote breastfeeding [14,15]. However, it would be a lengthy process to change the law and could impose additional economic costs on the government or companies. A more achievable goal is the encouragement of more breastfeeding-friendly workplace environments and the promotion of breastfeeding policies.

### Effect of worksite

This study found that breastfeeding-friendly policies can significantly affect whether office (white collar) workers start or continue to breastfeed after returning to work. Breastfeeding policies also influence the breastfeeding behavior of fab (blue collar) workers, but the effect is not as significant. The low percentage of those starting or continuing to breastfeed was partly because of an inconvenient working environment. Even when the manufacturer provided a lactation room and allowed one hour for milk expression, fab workers found it difficult to make the most of these measures: fab workers needed time to take off and put on their clean room suit, and it could take 10–15 minutes to travel between the very large cleaning room and the lactation room, leaving limited time for milk expression. This might help explain why the percentage of fab workers starting or continuing to breastfeed was low. Another possible reason is that women who breastfeed were more likely to find out about and paid attention to what benefits were available. Since the study was a cross-sectional study, it was our research limitation to find out the causality.

Research has also found that blue-collar worker participation in worksite health promotion programs is lower than that for white-collar workers [16-18]. This does not necessarily mean that blue-collar workers are less concerned about health issues, but rather it may reflect their own difficult circumstances, such as the lack of a supervisor's social support. For blue-collar workers, supervisors function as gatekeepers who control a worker's access to health promotion activities. For instance, to keep production lines moving, supervisors may refuse to allow workers to attend programs on company time [19]. This study also found that the group leader and the manufacturer's performance bonus system may reduce a woman's willingness to start or continue breastfeeding in the workplace. If a group leader disagrees with breastfeeding but the mother continues to breast pump during work hours, it will affect her work performance and bonus. Even if a mother sacrifices her bonus, her performance can affect the group's overall performance and collective bonus, such that the resulting negative working atmosphere could force a mother to choose between breastfeeding and her job.

### Worksite breastfeeding promotion

This study found that breastfeeding policies and facilities had a smaller effect on breastfeeding of fab workers than office workers. It also revealed that breastfeeding is not valued in the workplace. These findings were particularly relevant for Asian countries where maternity leave is short and most women return to work in the immediate post-partum period to full-time jobs where they have little direct control over their work environment. One survey found that most employers would be willing to institute policies that facilitate breastfeeding or breast pumping in the workplace. However, these employers saw little value to their business of supporting breastfeeding. [20] Another survey found that, while employers knew about the benefits of breastfeeding for mothers and children, they did not place a high priority on providing breastfeeding support [21]. For worksite breastfeeding promotion programs to be effective, they must involve not only the mothers, but also the employers and fellow workers.

## Conclusion

Some researchers opine that a mother's intention and breastfeeding knowledge are the key factors affecting breastfeeding behavior [22,23]. But intention or knowledge alone is not sufficient to overcome barriers to breastfeeding. We found that poor breastfeeding rates among blue-collar mothers in a company with breastfeeding-friendly policies was, at least in part, related to an inconvenient working environment. Galtry found that being blue collar or lower income generally implies lower skill requirements, less flexibility and even less protective working rights [24]. American Academy of Pediatrics Section on Breastfeeding indicated that exclusive breastfeeding for six months would lower the risk of morbidity and mortality of infants [25]. It is necessary to have more comprehensive breastfeeding-friendly measures in the workplace. The fab workers described in this study need more coordinated measures to support them in continuing to breastfeed after returning to work. Before criticizing women who discontinued breastfeeding, it is necessary to consider the environmental obstacles that mothers face and act to overcome them. Therefore, efforts at promoting breastfeeding need to deal with the mothers multiple enabling aspects, such as their socio-economic, cultural, and psychological conditions.

## Competing interests

The author(s) declare that they have no competing interests.

## Authors' contributions

Y. C. C. conducted the study, synthesized analyses and wrote the drafts of the article.

Y. C. W. undertook statistical analyses.

W. C. C. supervised the study and led the writing.

All authors helped to conceptualize ideas, interpret findings, and review drafts of the manuscript. All authors read and approved the final manuscript.

## Pre-publication history

The pre-publication history for this paper can be accessed here:



## Supplementary Material

Additional File 1 The file is a word file of the original questionnaire. Invitation and subject's information were not included. Click here for file

## Figures and Tables

**Table 1:** Characteristics of study subjects

	No. (%)
Age (year)	
20–24	066 (06.6)
25–29	419 (42.0)
30–34	406 (40.7)
≥35	107 (10.7)
Mean ± SD	29.8 ± 3.7
Maternity leave (day)	
<56 days	114(11.5)
56–60 days	835(84.0)
> 60 days	045(04.5)
Mean ± SD	56.8 ± 10.7
Breastfeeding initiation*	
Yes	587 (66.9)
No	291 (33.1)
Breastfeeding duration	
< 1 month	367 (62.5)
1–2 month	127 (21.6)
> 2 month	093 (15.8)
Education*	
≤ High school	512 (51.6)
≥ College	480 (48.4)
Number of children*	
1	432 (43.3)
2	441 (44.3)
≥3	124 (12.4)
Gender of child*	
Boy	504 (50.9)
Girl	487 (49.1)
Worksite	
Fab	819 (82.1)
Office	179 (17.9)
Shift Work	
No	228 (22.8)
Yes	770 (77.2)
Flextime	
Yes	282 (29.4)
No	676 (70.6)

* missing values excluded.

**Table 2:** Breastfeeding behavior of respondents by socio-demographic and working characteristics

	Never breastfed	Ever breastfed		
			
		Only during maternity leave	Continued after returning to work	Chi-square P
		
	n (%)	n (%)	n (%)	
Total*	291 (33.1)	494 (56.3)	93 (10.6)	
Age, years*				
< 30	123 (28.5)	275 (63.6)	34 (07.9)	<0.001
≥30	168 (37.7)	219 (49.1)	59 (13.2)	
Education*				
≥ College	99 (23.7)	251 (60.2)	67 (16.1)	<0.001
≤ High school	188 (41.2)	242 (53.1)	26 (05.7)	
Children's age				
<1 yr	45 (21.1)	133 (62.5)	35 (16.4)	<0.001
1–3 yr	164 (32.6)	291 (57.7)	49 (09.7)	
>3 yr	82 (50.9)	70 (43.5)	9 (05.6)	
Worksite				
Office	29 (18.8)	80 (52.0)	45 (29.2)	<0.001
Fab	262 (36.2)	414 (57.2)	48 (06.6)	
Shift work				
No	46 (24.1)	98 (51.3)	47 (24.6)	<0.001
Yes	245 (35.7)	396 (57.6)	46 (06.7)	
Flextime *				
Yes	69 (28.6)	126 (52.3)	46 (19.1)	<0.001
No	210 (34.8)	350 (58.1)	43 (07.1)	
Employment, years *				
≤6	130 (28.4)	276 (60.4)	51 (11.2)	<0.001
7–9	81 (31.2)	150 (57.7)	29 (11.1)	
≥10	80 (49.7)	68 (42.2)	13 (8.1)	
Awareness of lactation rooms *				
Yes	173 (27.5)	371 (59.0)	85 (13.5)	<0.001
No	117 (47.2)	123 (49.6)	8 (03.2)	
Awareness of breast pumping breaks *				
Yes	49 (30.8)	78 (49.1)	32 (20.1)	<0.001
No	238 (33.5)	412 (58.0)	60 (08.5)	

* missing values excluded.

**Table 3:** Factors related to breastfeeding by multivariate logistic regression

	Ever breastfed*			Continued breastfeeding after returning to work†		
	
	OR‡	(95% CI)	P value	OR‡	(95% CI)	P value
Age, years						
< 30/≥ 30	1.37	(0.99–1.89)	0.057	0.69	(0.40–1.23)	0.211
Education						
> College/≤ College	1.59	(1.12–2.17)	0.008	1.03	(0.57–1.87)	0.926
Children's age						
<1 yr/>3 yr	1.34	(0.54–3.31)	0.529	2.53	(1.55–4.14)	<0.001
1–3 yr/>3 yr	0.85	(0.36–1.99)	0.701	1.64	(1.10–2.42)	0.014
Worksite						
Office/Fab	2.33	(1.40–3.87)	0.001	4.32	(2.30–8.11)	<0.001
Employment, years						
≤6/≥10	1.77	(1.17–2.67)	0.007	1.14	(0.53–2.43)	0.745
7–9/≥ 10	1.91	(1.25–2.94)	0.003	0.83	(0.42–2.02)	0.826
Awareness of lactation rooms						
Yes/No	1.60	(1.14–2.24)	0.006	2.71	(1.19–6.15)	0.017
Awareness of breast pumping breaks						
Yes/No	0.87	(0.59–1.28)	0.474	2.68	(1.57–4.58)	<0.001

Note: OR=odds ratio; CI=confidence interval.

*Women who never breastfed as reference group.

† Women who only breastfed during maternity leave as reference group.

‡ Adjusted for all other variables in the table.

**Table 4:** Effects of awareness of policy on breastfeeding behaviors

	Ever breastfed*			Continued breastfeeding after returning to work†		
	
	OR‡	(95%CI)	P value	OR*	(95%CI)	P value
**Stratified by worksite**						
Awareness of lactation room	Fab‡					
Yes/No	7.88	(2.36–26.32)	0.001	2.06	(0.78–5.40)	0.143
Awareness of breast pumping breaks						
Yes/No	0.84	(0.56–1.26)	0.399	2.15	(1.09–4.26)	0.028
Awareness of lactation rooms	Office ‡					
Yes/No	2.83	(0.99–8.06)	0.052	6.53	(1.33–32.13)	0.021
Awareness of breast pumping breaks						
Yes/No	1.14	(0.33–3.95)	0.833	4.91	(1.79–13.46)	0.002
**Selected by awareness of policy**						
Worksite	High awareness of policy ‡§					
Office/Fab	2.38	(0.65–8.71)	0.189	8.65	(2.48–30.18)	0.001

*Women who never breastfed as reference group.

† Women who only breastfed during maternity leave as reference group.

‡Adjusted for mother's age, education, employment and children's age.

§Only women who both knew about lactation rooms and breast pumping breaks in the model.
