# Supplementary material for: Effects of work-related factors on the breastfeeding behavior of working mothers in a Taiwanese semiconductor manufacturer: a cross-sectional survey
Source: BMC Public Health. 2006 Jun 21;6:160. doi: 10.1186/1471-2458-6-160 (PMC1538587; doi:10.1186/1471-2458-6-160)
Supplement: Additional File 1 — The file is a word file of the original questionnaire. Invitation and subject's information were not included. [file 1471-2458-6-160-S1.doc]

**Work ID□□□□□□□□□ Name Tel number**

# Please answer the following questions on how you nursed your most recently born child

**Information about the most recently born child**

1.Child’s birth date yy/mm

2.Child’s birth weight kg

3.Child’s order □1.The first one □2. The second one □3. The third one □4.others

4.Child's gender □1.Male □2.Female

5.Is he/she a premature baby? □1.Yes □2.No

6.Did you want to breastfeed him/her after the baby was born?

□1.strong intention □2.have intention □3.no intention

7.How long was your maternity leave? day

**Next, we would like to know about your work and nursing status after returning to work**

8.How long did you spend on your daily commute before the most recently born child was one year old?

□1.in one hour □3.in three hours

□2.in two hours □4.over three hours

9.Did you do shift work after you returned to work? □1.Yes □2. No

10.Did you need any substitute when you left your position during working hours?

□1.almost □2.almost not

11.Before the child’s one year birthday, how often were you able to see your child?

- 1.almost everyday.
- 2.not everyday, at least two days a week.
- 3.not everyday, about two days a month.
- 4.less than two days a month.
- 5.others, explanation

# Next, we would like to know about your workplace's breastfeeding policy and your infant's feeding status

12.Did you know about the lactation room in your company?

- 1.Yes □2.No

12a. Is the location convenient for you?

□1.Yes □2.OK □3.No □4. Never us, no idea

12b.Have you ever used the lactation room?

□1.Yes *(jump to13)* □2.No (please answer 12c)

12c.Why did you not use it?

1.Not breastfeeding after return to work

2.Not pumping breast milk, only breastfeeding at home

3.Found another place to pump

4.other reason, explanation ____________________

13.How do you feel about “the lactation room” (multiple-choice)

- 1.very good
- 2.no idea
- 3.space is too small
- 4.the location was too far, need more lactation rooms
- 5.the location is not convenient, because they take care the sick people at the same place.
- 6 provide some facilities such as hospital grade breastpump or electronic steam sterilizer
- 7.other suggestion, explanation

14.Did you know about pumping breaks? 1.Yes □2.No

# 15.How do you feel about the pumping breaks?( multiple-choice)

- 1. very good
- 2. no idea
- 3. no promotion, I don’t know about the rule
- 4. not enough, I wish it could be longer for (hour)
- 5. uncomfortable to use it because of the work situations and concerns
- 6.bebause the work pressure, I seldom use the break or can’t take the break. Only breastfeeding at home
- 7.other, explanation

16.Did you ever breastfeed your most recently born child?

□1.Yes(Please answer question 16.ab) 2.□No *(jump to 18)*

16a. Do you breastfeed now? □1.Yes □2.No

16b. How long did you breastfeed your most recently born child?

Days, or Weeks, or months

16c.How did you breastfeed?

□1.breast-feeding □2.bottle-feeding

□3.combine breast and bottle feeding □4.others, explanation

17. Did you have any breast pumping experience at your workplace after your most recently born child was born?

- 1.Yes(please answer the following □2.No *(jump to 18)*

17a.Did you use the breastfeeding break?

□1.Yes *(jump to 17c)*  □2.No ( please answer17b.)

17b. Why not? ( multiple-choice)

□1.I think my boss may disagree about it

□2.I don’t think my colleagues agree or support it

□3.I don’t think I should use it

□4.Others, explanation

17c. Did you still pump your breast milk now?

□1. Yes *（jump to 18）* □2.No（please answer the following）

17d.When did you stop to pump breast milk?

When the child was months old

17e.Why did you stop? (multiple-choice)

□1.My child was old enough

□2.Less breast milk or no breast milk

□3.My breast milk was not good enough

□4.Take the night shift

□5.Increasing of my work loading, no time to pump

□6.Other reason, explanation

# Next, we would like to know about your social support

18. Did your husband agree that you should breastfeed the child after your **most recently born** was born?

□1. Yes □2.No □3.No opinion □4.other

19.Who was the major caregiver for the most recently born child?___________

Did he/she agree you to breastfeed?

□1. Yes □2.No □3. No opinion □4.other

20.Did your direct supervisor agree with his/her direct subordinates' decision to pump breast milk in the workplace?

□1. Yes □2. No comment □3.Depend on the situation □4.No □5. I don’t know what he thinks

21.Did your direct supervisor take action to support mothers to pump breast milk in the workplace? (such as coordinate someone to help the mothers leave to pump their breast milk)

□1. Yes □2. Depend on the situation □3.No □4. I have no idea about their opinion

22.Did most of your colleagues (defined as people working with you) agree that mothers can pump their breast milk in the workplace?

□1.Most of them agree (or no opinion)

□2.Some of them agree, some of them do not

□3.Most of them don’t agree

□4.I have no idea about their opinion

23.Would they like to take action to support the mothers?

（such as: substitute the mother's work for 4 months when she leaves to pump breast milk, take the night shift）

- 1.Most of them would like
- 2.Some of them would, some of them would not
- 3.Most of them would not
- 4. I have no idea about their opinion

24.Did you agree that mothers could pump their breast milk at workplace?

□1.Yes □2. Depend on the situation □3.No

25.Would you like to take action to support them?

（such as: substitute the mother's work for 4 months when she leaves to pump breast milk, take

the night shift）

□1.Yes □2. Depend on the situation □3.No

26. Did you know anyone who used to pump her breast milk when you delivered your most recently born child?

□1. Yes □2.No*（jump to 27）*

27.Would they like to give any support about breastfeeding?

□1.Yes □2.No

**Your information**

28. Educational level □1.High school □2.College □3. Graduate school and over

29. Job position_______
